# 

**Published:** 1898-07

**Authors:** 


### CONTENTS:

### SELECTIONS.

Article I.—Dental Anatomy. Dr. H. A. Thompson ........... 97
" II.—Condensation of Gold Fillings. Dr. A. G. Smith..... 103
" III.—Care and Treatment of Children's Teeth. J. W. Paul, D. D. S.......................................... 107
" IV.—Adapting Artificial Dentures. Dr. A. O. Hunt....... 109
" V.—Reproduction of Gum Tissue. George T. Carpenter, M. D., D. D. S. .................................... 110
" VI.—Painless Dentistry. Dr. Clyde Payne................ 114
" VII.—Dental Ethics. Prof. S. H. Guilford................ 115
" VIII.—Advertising. H. C. Sexton, D. D. S.................. 120
" IX.—Gingivitis and its Relation to Crown Work. S. B. Palmer, M. D. S.......................................... 129

### COMMUNICATED.

Seventh Annual Commencement, Dental Department, Uuiversity of Buffalo ............................................... 137

### EDITORIAL, ETC.

Questions Given by Maryland State Board of Dental Examiners, May, 1898 ................................................ 138

### MONTHLY SUMMARY.

Cohesive and Non-Cohesive Gold.... ............................ 141

Death from Hodgkins' Disease.................................. 142

A Student's Damage Suit.......... .............................. 143

Foreign Doctors and Dentists in Italy......................... 144

